# An Ethylmethane Sulfonate Mutant Resource in Pre-Green Revolution Hexaploid Wheat

**DOI:** 10.1371/journal.pone.0145227

**Published:** 2015-12-17

**Authors:** Amandeep K. Dhaliwal, Amita Mohan, Gaganjot Sidhu, Rizwana Maqbool, Kulvinder S. Gill

**Affiliations:** Department of Crop and Soil Sciences, Washington State University, Pullman, Washington, 99164, United States of America; Institute of Genetics and Developmental Biology, CHINA

## Abstract

Mutagenesis is a powerful tool used for studying gene function as well as for crop improvement. It is regaining popularity because of the development of effective and cost efficient methods for high-throughput mutation detection. Selection for semi-dwarf phenotype during green revolution has reduced genetic diversity including that for agronomically desirable traits. Most of the available mutant populations in wheat (*Triticum aestivum* L.) were developed in post-green revolution cultivars. Besides the identification and isolation of agronomically important alleles in the mutant population of pre-green revolution cultivar, this population can be a vital resource for expanding the genetic diversity for wheat breeding. Here we report an Ethylmethane Sulfonate (EMS) generated mutant population consisting of 4,180 unique mutant plants in a pre-green revolution spring wheat cultivar ‘Indian’. Released in early 1900s, ‘Indian’ is devoid of any known height-reducing mutations. Unique mutations were captured by proceeding with single M_2_ seed from each of the 4,180 M_1_ plants. Mutants for various phenotypic traits were identified by detailed phenotyping for altered morphological and agronomic traits on M_2_ plants in the greenhouse and M_3_ plants in the field. Of the 86 identified mutants, 75 (87%) were phenotypically stable at the M_4_ generation. Among the observed phenotypes, variation in plant height was the most frequent followed by the leaf morphology. Several mutant phenotypes including looped peduncle, crooked plant morphology, ‘gritty’ coleoptiles, looped lower internodes, and burnt leaf tips are not reported in other plant species. Considering the extent and diversity of the observed mutant phenotypes, this population appears to be a useful resource for the forward and reverse genetic studies. This resource is available to the scientific community.

## Introduction

Functional genomic resources are important to determine function of the genes particularly that control agronomic traits. Gene alteration through mutagenesis is an important approach to generate such functional resources. Ethylmethane Sulfonate (EMS) has been the mutagen of choice in forward genetics approach to generate mutagenized populations in a wide array of species. EMS is an alkylating agent that causes mainly point mutations due to the addition of an alkyl group on guanine resulting in transition of GC to AT. EMS induces mutations creating allelic versions of genes resulting in phenotypes of varying intensity [1]. The forward genetic approach enables identification of agronomically desirable phenotypes that can be exploited in breeding programs. In wheat, the forward genetics approach has been successfully used to identify mutants of agronomic importance including imidazolinone tolerance [2], waxiness, and grain hardness [3] (http://wheat.pw.usda.gov/GG2/Triticum/wgc/2008/).

A number of publicly available mutant populations have been developed in various cultivars of bread wheat (*Triticum aestivum* L.) but their utility has been limited to only few traits with discernible phenotypes and to genes with relatively well-characterized functions. Examples of such genes include waxy in elite cultivars Express [4], Cadenza [5], and Ventura [6]; puroindolines in Australian soft-textured grain cultivar QAL2000 [6] and Alpowa [7]; *Wheat Kinase Start* (*WKS*) *1*, *WKS2*, and *Starch Branching Enzyme* (*SBE*) *IIa* in breeding line UC1041+Gpc-B1/Yr36 [8], *photoperiod* (*Ppd*)-*D1*, *Rubisco activase A* and *B* [9], and *starch synthase II* [5]. Depending upon the EMS concentration, detection approach, and the target gene, the reported mutation frequency in hexaploid wheat ranged from 1/47 kb to 1/12 kb [6,9]. This frequency is expected to increase significantly with the adoption of more sensitive detection methods [10]. The available mutant populations are in the post-green revolution cultivars carrying ‘reduced height’ (*rht*) genes that may be lacking many useful alleles lost during selections leading to the green revolution. Further, with the changing environmental conditions and the ill-effects of the currently used *rht* dwarfing genes on a number of plant attributes including coleoptile and first leaf length, and primary and secondary roots and root biomass, it is important to develop a mutant resource in a non-*rht* genotype [11,12]. The objective of our study is therefore to generate and characterize an EMS mutant population in ‘Indian’, a tall hexaploid wheat cultivar released in early 1900s.

## Materials and Methods

### Plant material

A soft white spring hexaploid wheat cultivar Indian (CItr 4489) [13] with an average height of 103 cm, six tillers, and semi-awned spikes was used for mutagenesis. Being the tallest among the older cultivars in our world wheat collection [14], it lacks any of the known dwarfing mutations and possibly contains valuable alleles that got selected out during breeding over the decades.

### EMS mutagenesis

Each of the initial experiment to optimize EMS concentration was carried out using 200 seeds of cultivar Indian. EMS concentrations of 0, 10, 20, 30, 40, and 50 mM were tested to select 40 mM concentration (see results) to carry out the subsequent experiment using 600 gm seed of cultivar Indian.

For mutagenesis, 200 seeds were soaked in 40 ml of de-ionized water (3,000 ml for 600 gm of seed) in a flask that was kept on a benchtop shaker (Barnstead Labline—MaxQ 3,000), for four hours with gentle shaking at room temperature, with hourly change of water. After four hours, the water was replaced with the appropriate concentration of EMS solution and the flasks were kept on the shaker for 18 hours. The EMS solution was removed and the seeds were washed twice with 100 mM sodium thiosulphate solution for 30 minutes each. Seeds were dried for 24 hours on tissue papers at room temperature before planting at a very high density (to facilitate growth of only single tillers) in large plastic trays with holes containing the professional growing mix (Sunshine LC1 mix). The plants in the trays were grown in the greenhouse (Wheat Growth Facility, WSU) with 16 hours light, and 22°C temperature during the day and 18°C during the night in 2010. The main tiller of each of the surviving 4,180 M_1_ plants was individually harvested and thrashed. In 2011, single M_2_ seed from each of the M_1_ plants was planted in the greenhouse. The M_3_ seed from each of the surviving M_2_ plants was bulk harvested and planted in six feet rows at the Spillman Agronomy Farm, Pullman, WA in 2011. For the selected 86 mutants, single plant showing the best mutant phenotype was harvested. For the remaining population, M_4_ seed of each line was bulk harvested and stored at -20°C. The M_4_ generation of the selected mutants was grown in three feet rows at the Spillman Agronomy Farm, Pullman, WA in 2012.

### Greenhouse and field evaluation of the mutant population

Detailed phenotypic data at the tillering and maturity stages was collected on 1,902 of the 4,180 greenhouse grown M_2_ plants of the mutant population. At the tillering stage, plants were scored for the plant architecture, number of tillers, and leaf and stem morphology. Data on plant height and tiller uniformity was collected at maturity along with the number of internodes, and stem and spike morphology. The remaining ‘fertile’ 1,561 M_2_ plants from the population were grown in 2013 for seed multiplication.

With the objective to: a) study the stability of the mutant phenotype; and b) multiply the seed; approximately 60 M_3_ seeds of each of the 1,902 M_2_ plants were planted in field. Four rows were planted in each plot with a row-to-row and plot-to-plot spacing of one foot. Each row represented progeny of a single M_2_ plant. For comparison, control cultivar Indian was planted after every 19 mutant rows. Starting at the seedling stage, M_3_ plants were evaluated throughout the growing season for phenotypic differences in comparison to the wild type Indian. Leaf related phenotypes including leaf spots, leaf width, and leaf color were more obvious at the tillering stage. Tiller number, peduncle, stem, spike, and spikelet related morphologies were scored during later stages of the plant development. Data on plant height was collected on all the 1,902 plants of this population. The selected M_3_ mutants were grown again in three-feet rows in 2012 with the similar field layout to study stability of the mutations. M_4_ plants were evaluated similarly throughout the growing season for phenotypic differences in comparison to the wild type Indian. The remaining 1,561 of the 4,180 M_3_ plants were seed-increased by growing in three-feet rows with a similar field layout in 2014.

## Results

### Optimization of EMS concentration

To determine a suitable concentration of EMS for the mutagenesis of cultivar Indian, 200 seeds of each of the six treatments (0, 10, 20, 30, 40, and 50 mM) were planted in cones in the greenhouse and the M_1_ plants were taken to maturity. Compared to 195 plants for the control (no EMS), 50 mM showed the least number of survived M_1_ plants and the highest proportion of the plants showed aberrant phenotype. The M_2_ plants from each of the treatment were planted in the field. The plants treated with 50 mM concentration showed a very high proportion of sterility compared to the 40 mM treatment. Number of plants showing aberrant phenotype in the 40 mM treatment were higher, thus, 40 mM concentration was used for further experiments.

### Identification and evaluation of mutations over generations

**Fig 1:**
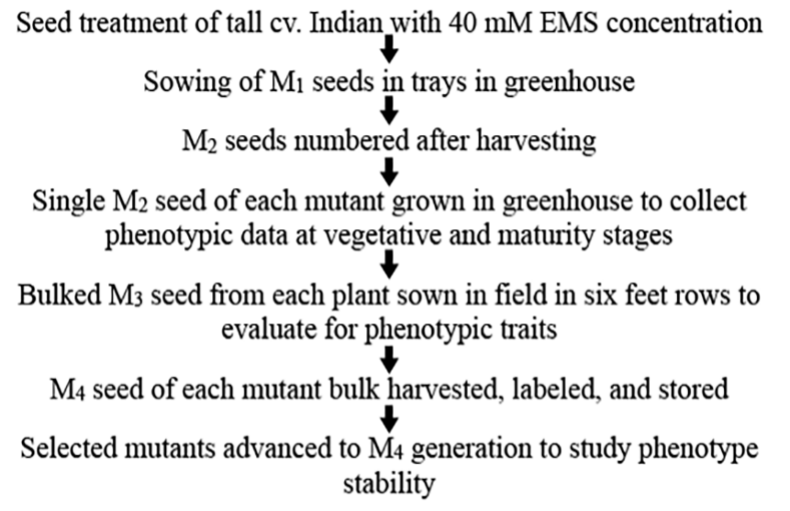
Development of EMS mutagenized population in a pre-green revolution tall cv. Indian and its phenotypic analysis over generations and growing conditions.

**Fig 2:**
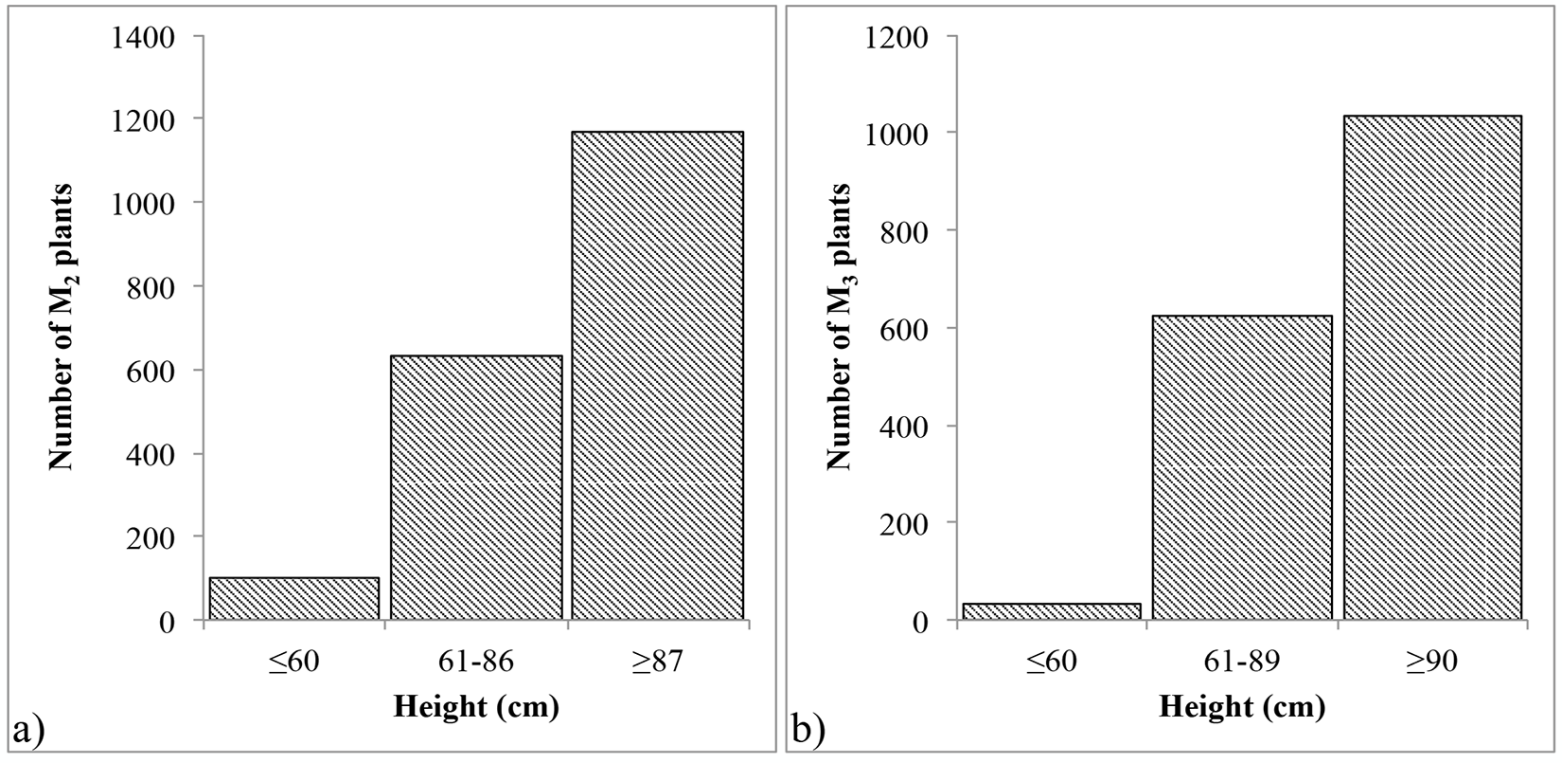
Distribution of plant height of (A) M_2_ plants in greenhouse and (B) M_3_ plants in field. Height of control cv. Indian was 102 cm (87–113 cm) in greenhouse and 106 cm (90–125 cm) in field.

The procedure to develop and evaluate mutant plants over generations is given in Fig 1. About 13% (278) of the 2,180 M_2_ seeds either didn’t germinate or produced nonviable plants. Viable plants (1,902) were compared to control Indian to identify the plants showing a mutant phenotype. Compared to control Indian, 453 of the 1,902 M_2_ plants were putative mutants that showed phenotypic differences for the plant traits other than height and peduncle. Of these putative mutants, 155 showed uniform tillers compared to that of non-uniform tiller growth in control Indian and 141 showed sturdier stem (Table 1). With an objective to identify height mutations in pathways other than giberrellic acid (GA), the entire population was evaluated to select plants with height comparable to that of the currently grown wheat varieties. Compared to the average plant height of 103 cm (range of 87–113 cm) for the control Indian in the greenhouse, 733 plants showed plant height less than 87 cm, thus were selected as putative height mutants. Among these plants, 100 showed a height of ≤60 cm (dwarf) and 633 from 61 to 86 cm (semi-dwarf). 157 M_2_ plants were taller than the control with height ranging from 114–137 cm. The remaining 1,012 M_2_ plants showed plant height ranging from 87–113 cm, similar to that of the control Indian (Fig 2A).

**Table 1:** Phenotypic data collected on single M2 plants in greenhouse at different developmental stages.

Phenotypic Trait	Observations	Number of Plants
Habit	Bushy or grassy	15
Leaf	Broad and dark green	12
	Flag leaf close to spike	5
Tiller	Uniculm	15
	>9 tillers	20
	Uniform tillers	155
Peduncle	Wavy	1
Stem	Sturdy	141
	Bent	16
Node	Swollen	24
Internode	Thick	3
Spike length	Long	7
	Short	3
Spike	Relaxed	3
	Compact	1
	Deformed	2
	Awned	10
Plant Maturity	Late	20

**Fig 3:**
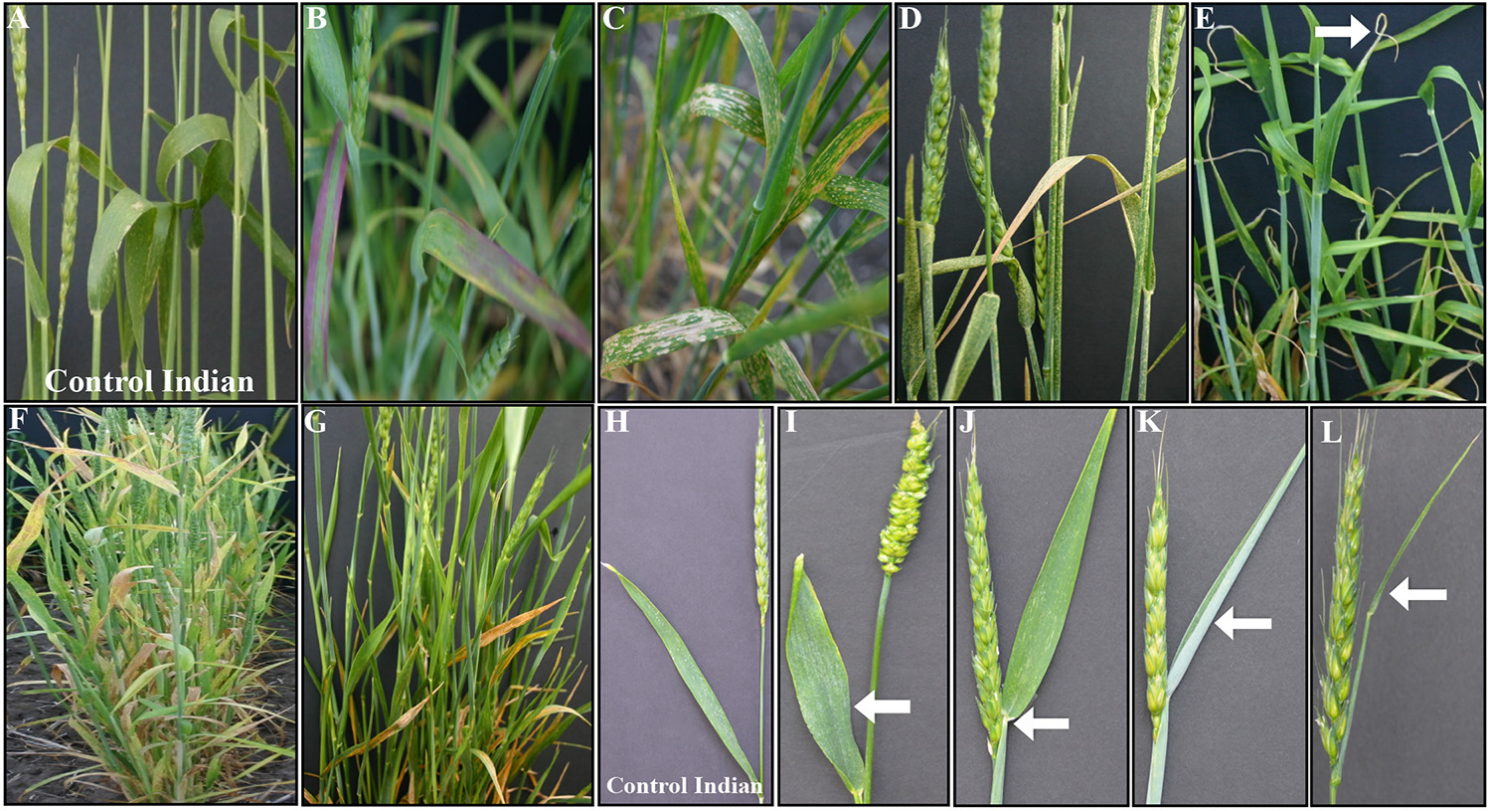
Phenotypes of M_3_ mutants for leaf color and morphology (B-G) and flag leaf morphology (I-L). Leaf phenotypes include anthocyanin pigmentation (B), chlorotic spots (C), lesion mimic (D), burnt leaf tips (E), yellow-green (F), and dark green leaves (G) compared to control Indian (A); and flag leaf morphology includes broad and short leaf (I), flag leaf close to lower part of spike (J), silver-green and rolled flag leaf (K), and short peduncle with thin sheath and flag leaf (L) compared to control Indian (H). Changes in mutant phenotype compared to control Indian are marked with arrow.

**Fig 4:**
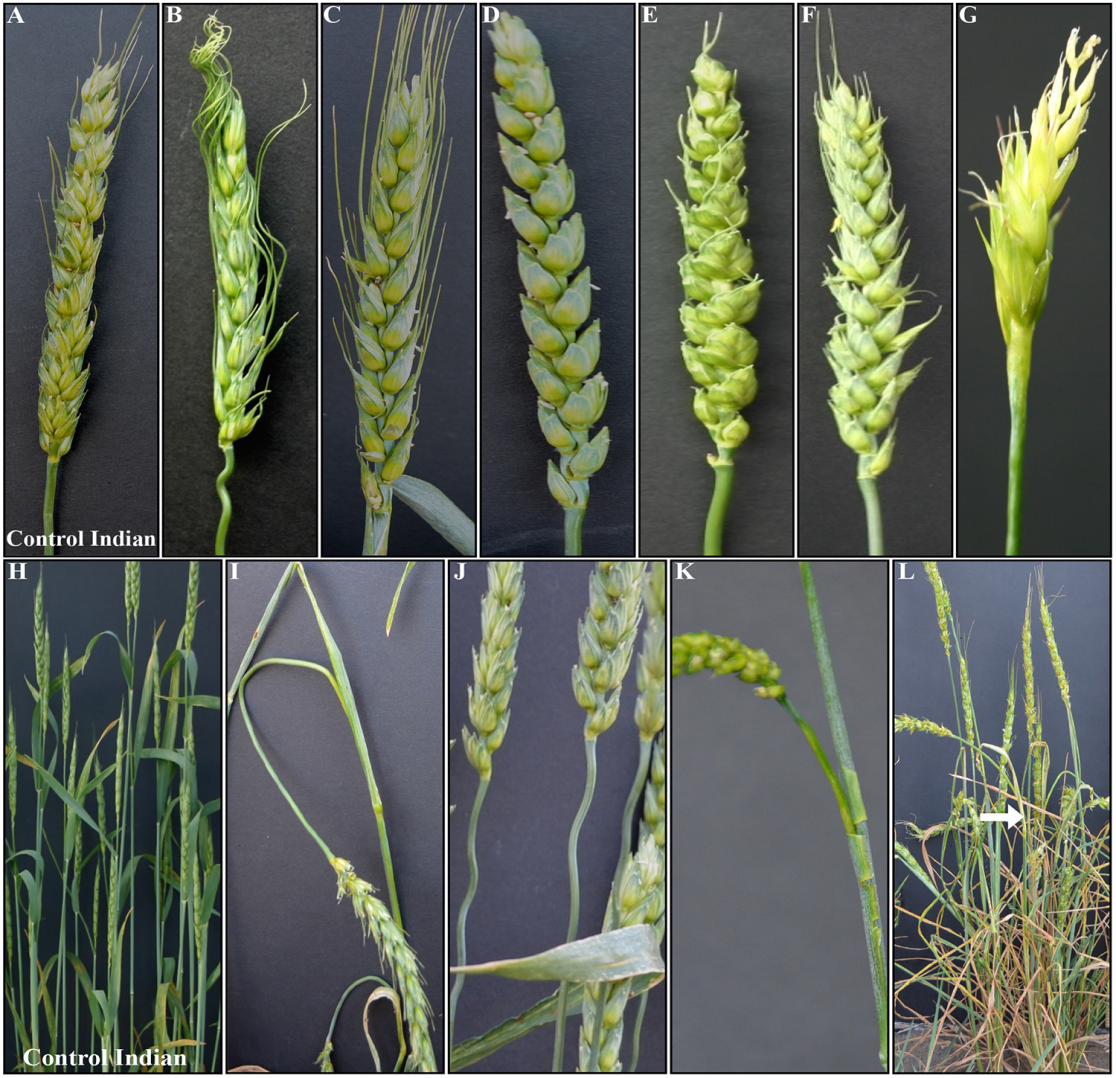
Spike and peduncle morphology of mutant plants. Compared to medium sized awns in control Indian (A), spike with large awns (B, C), awnless (D), small awns (E), spikelet morphology (E, F), and top spikelets sterile (G). Variations in peduncle morphology include bent (I), wavy (J), shredded flag leaf sheath (K), and crooked phenotype with crinkled leaf and peduncle (L) compared to control Indian (H). Phenotypic variations compared to control Indian are marked with arrow.

Of M_3_ progeny of the 1,902 M_2_ plants grown in the field as head-rows, 1,690 survived. The progeny of remaining 212 either didn’t germinate or were nonviable. A row was considered to be a phenotypic mutant if two or more plants showed the mutant phenotype in comparison to the wild-type Indian. Using this criterion, 240 M_3_ rows were identified to be carrying phenotypic mutants for the traits other than height. Among these, leaf morphology including leaf shape, angle, and color, was the most frequent variation with 69 M_3_ lines followed by 66 M_3_ lines for early flowering (Table 2). Leaf morphology included spots of varying sizes with a range in phenotypic severity (lesion mimic) (Fig 3B and 3C); leaf color of dark green, yellow-green, silver-green, and purplish pigmentation (Fig 3); and upright leaf angle, curled, and crinkled leaves (Table 2). Of the 37 mutants for spike morphology (Fig 4A–4G and Table 2), plants with longer awns, compared to medium awns in control Indian, were the most frequent type. Among mutant plants for stem morphology, sturdy stem was the predominant phenotype. The least frequent variations included iojap, uniculm, lazy, and bent nodes (Table 2). Both flag leaf and peduncle showed variations for size and growth pattern (Fig 3I–3L). Among 240 mutants other than the height mutants, 59 showed more than one mutations either for leaf color, leaf shape, stem thickness, early flowering, bent nodes, awned/awnless spike, deformed spike, and uniculm.

**Table 2:** Phenotypic data on M_3_ rows in field at different developmental stages.

Phenotypic Trait	Observations	Number of plants
Leaf angle and shape	Upright	11
	Curled	7
	Crinkled	1
Leaf color	Dark green	14
	Silver green	10
	Yellow green	7
	Anthocyanin	2
	Lesion mimic	17
Stem morphology	Sturdy	17
	Thin	5
Peduncle	Inverted	3
	Wavy	1
Spike morphology	Awned	30
	Compact	4
	Deformed	2
	Silver color	1
Others	Uniculm	15
	Lazy	1
	Bent nodes	1
	Grassy	18
	Iojap	7
	Early flowering	66

Compared to the average height of 105 cm with a range of 90 to 125 cm for the wild-type Indian, significant variation was observed among M_3_ mutants for the plant height in the field (Fig 2B). Height of 654 M_3_ lines was less than 90 cm of which 32 were considered to be dwarf with an average height of ≤60 cm and the remaining 622 were semi-dwarf with plant height ranging from 61 to 89 cm. Plant height of the remaining 1,036 M_3_ plants ranged from 90–125 cm similar to that of wild-type Indian.

The height of each mutant was compared between M_2_ and M_3_ generations. The control Indian has an average height of 103 cm [Coefficient of variation (CV) 9.3%] in M_2_ and 105 cm (CV 8.4%) in M_3_ generation. Considering average CV of 8.9% for control Indian, height of 662 M_2_ mutants remained same in M_3_ generation while 540 mutants showed decrease and 484 mutants showed increase in height.

**Fig 5:**
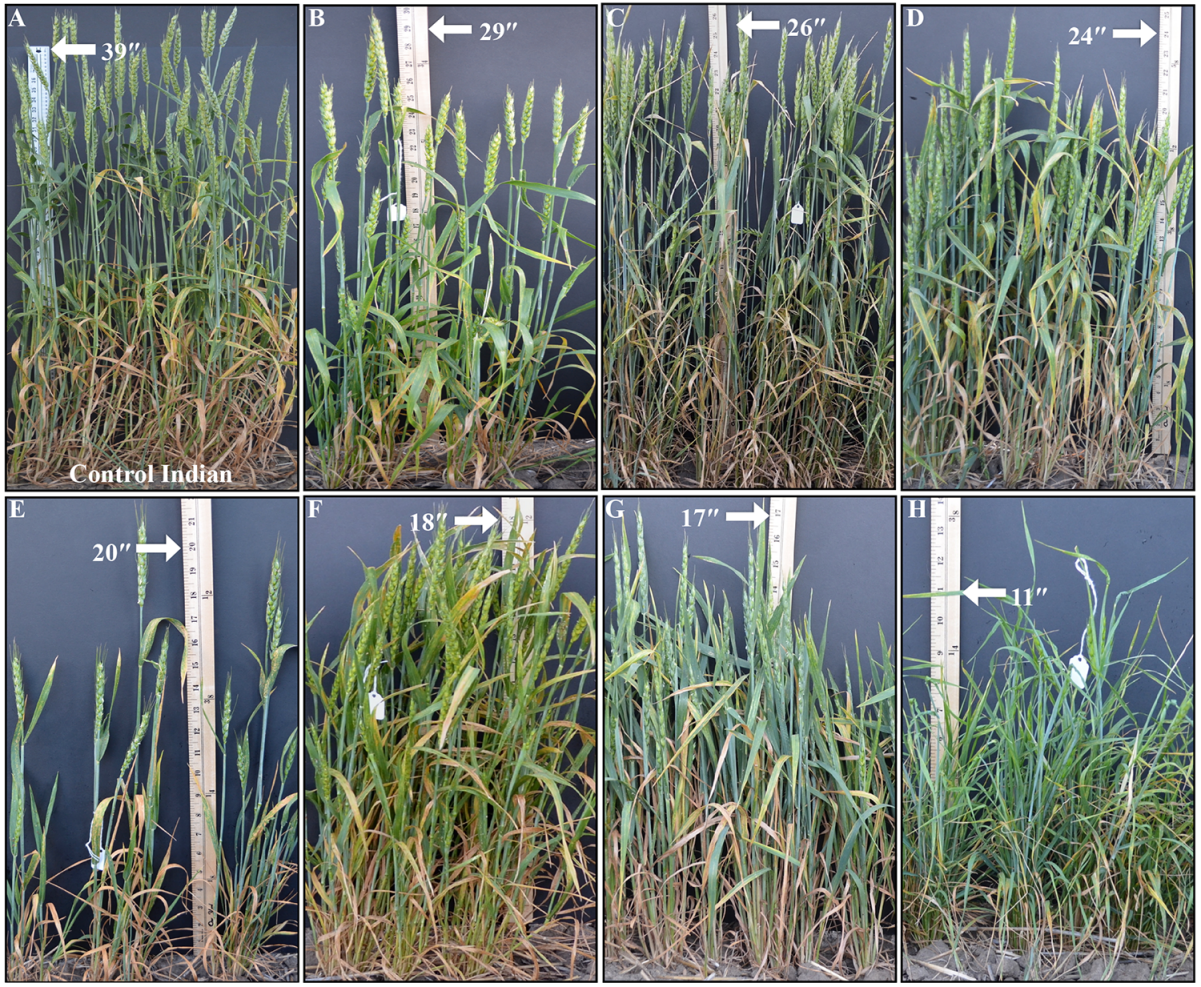
Comparison of M_4_ height mutants growing in three feet rows (B-H) with control Indian (A) grown in the same field conditions. Measuring scale is in inches and arrow indicates the height of mutants.

The M_4_ progenies of 86 of the above-mentioned mutants selected for their obvious phenotypes were grown as rows in the field for phenotype stability. Thirty-five of these mutants were for the traits other than plant height including variation for spike, leaf, stem, and peduncle. Nine of these mutants were ‘uniculm’, showing only a single tiller. The remaining 42 mutants showed variation in plant height ranging from 33 to 87 cm including the agronomically desirable height range of 60 to 80 cm as observed for the popular wheat varieties grown in the same field (Fig 5). Control Indian has average height of 105 cm (CV 2.6%) in M_4_ generation. Considering the average CV of 5.5% of control Indian over M_3_ and M_4_ generation, 26 of these 42 mutants showed reduction in plant height, eight showed an increase, and the remaining eight mutants showed no change in height.

### Cataloging of the stable mutations

Stability of mutations was evaluated by assessing the same mutations over generations to rule out the environmentally influenced phenotypes. From the selected 733 M_2_ mutants with height <87 cm, 168 either did not germinate or were nonviable in M_3_ generation but 258 showed height similar to that of the previous generation. For the remaining 307 putative mutants, the phenotype was not evident in M_3_ generation and their height was similar to that of the wild-type Indian. Plant height of these 307 putative mutants during M_2_ generation ranged from 40 to 86 cm. Therefore only 35% of the height mutants remained stable at M_3_ generation. Stability of the mutant phenotype after M_3_ generation was high. All selected 42 height mutants (see above) showed a height phenotype less than wild-type Indian in M_3_ and M_4_ (Table 3).

**Table 3:** Comparison of height across M3 and M4 generations compared to average height of control Indian within each generation.

Mutant	Plant Height (cm)	
	(M_3_)	(M_4_)
2001	63	69
2015	76	58
2038	70	62
2056	71	61
2061	65	61
2068	56	33
2105	75	77
2149	73	64
2157	65	77
2216	75	72
2259	77	68
2293	58	65
2310	65	52
2365	57	57
2465	65	61
2485	65	65
2570	69	75
2651	55	67
2669	50	59
2735	64	87
2752	76	83
2763	58	35
2838	39	50
2840	58	37
2897	73	62
2921	64	77
2967	64	55
3003	64	44
3086	70	54
3140	65	47
3167	62	79
3277	62	55
3446	50	42
3608	35	52
3718	80	78
3756	72	53
3884	64	55
3892	57	55
3939	76	49
3956	70	49
4041	40	72
4111	61	44
Indian	106	105

Stability of the mutants other than that for height was also tested over generations. Of the 453 plants identified to be mutants during the M_2_ generation, only 24 (5%) showed the same phenotype in M_3_ generation and the remaining 95% behaved as control Indian. Seven of these 24 mutants along with additional 37 M_3_ mutants selected on the basis of phenotypic traits and single tiller in the M_3_ generation were further grown in the field to test for the stability in the M_4_ generation. Of these, phenotype of 33 (75%) mutants was stable at this generation.

**Fig 6:**
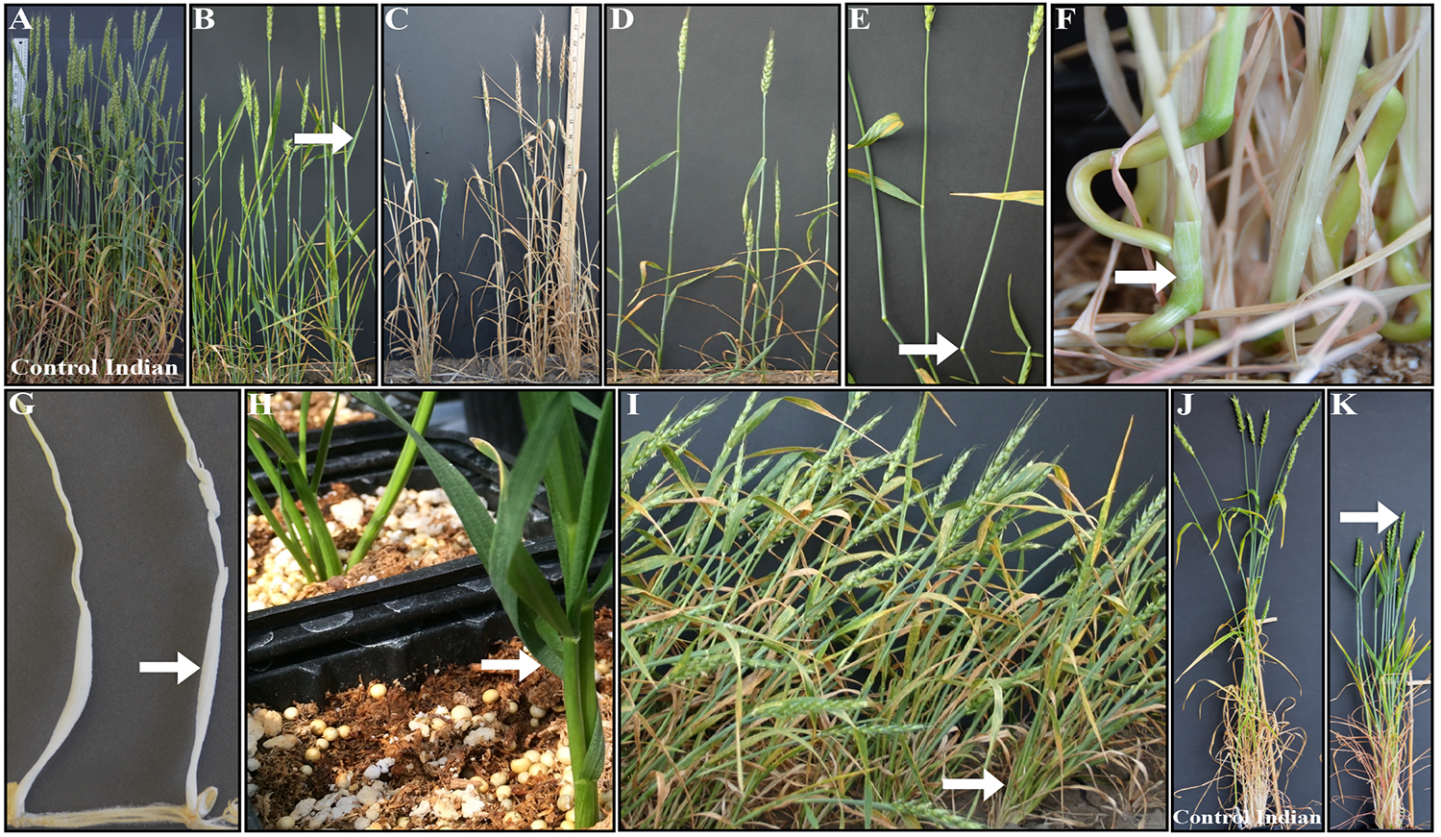
Phenotypic variations observed in mutants compared to control Indian (A, J). These variations include upright leaves (B), early senescence (C), single tiller (D), bent nodes (E), looped lower internodes (F), gritty coleoptile (G), leaf curled around stem base (H), lazy mutant (I), and uniform tillers and upright leaves (J). Phenotypic variations compared to control Indian are marked with arrow.

Overall, 75 mutants showing reliable and stable phenotype including height over generations were selected (Table 4). These included lesion mimic, yellow green leaves, dark green leaves, anthocyanin pigmentation, early senescence, awned, grassy, upright leaves, and uniculm mutants (Figs 3, 4 and 6 and Table 2). However, to the best of our knowledge, several of the mutants identified during this study were novel and have not been reported in other plant species (Table 5). These include ‘burnt leaf tips’ (Fig 3E), looped peduncle (Fig 4I), crooked plant morphology (Fig 4L), looped lower internodes (Fig 6F), and ‘gritty’ coleoptiles (Fig 6G). ‘Lazy’ mutant was also identified for the first time in hexaploid wheat (Fig 6I).

**Table 4:** Number of stable mutants for height, uniculm, and other phenotypes in M4 generation selected from M3 generation.

Phenotype	Generation	
	M_3_	M_4_
Height	42	42
Uniculm	9	7
Visible phenotypes	35	26

**Table 5:** Novel phenotypic mutants identified in hexaploid wheat.

Phenotype	Phenotype known in other species	References
Looped peduncle	No	
Looped lower internodes	No	
Burnt leaf tips	No	
Gritty coleoptile	No	
Crooked plant morphology	No	
Wavy peduncle	Barley	http://www.distagenomics.unibo.it/TILLMore/
Lazy	Maize, sorghum, rice	Dong et al., 2013, Ordonio et al., 2014, Li et al., 2007

### Analysis of stable height mutants

The stable 42 height mutants in M_4_ generation were analyzed for relative internode length in comparison to the plant height (Table 6). Percent internode was calculated as the contribution of each internode towards the total plant height. Compared to control Indian, the mutants differed significantly for the relative internode length (Table 6). About equal number of the height mutants showed an increase and decrease in internode 1 length. Relatively fewer plants showed a change in internode 2 or 3. Maximum number of mutants (25/42) showed reduction in internode 4. Twenty of these mutants along with two other mutants showed no visible 5^th^ internode.

**Table 6:** Contribution of internodes toward plant height in mutants compared to the control cv. Indian. Internode 1 corresponded to the peduncle.

Internode	Less than control		More than control		Equal to control
	Number of mutants	Percent reduction in internode	Number of mutants	Percent increase in internode	Number of mutants
1	14	17.8%	16	23.7%	12
2	8	24.1%	15	19.4%	19
3	1	20.3%	16	33.1%	25
4	25	49.0%	3	29.1%	14
5*	6	71%	-	-	14

* 22 mutants have no 5^th^ internode.

## Discussion

### Identification of mutations in pre-green revolution cultivar

Most of the mutant populations available in hexaploid wheat are developed in recently released cultivars carrying *rht* mutations. Because of the genetic uniformity created by the use of common *rht* mutations, and breeding and selection during the last few decades, the popular wheat varieties around the world have a narrow genetic base and probably are lacking valuable gene/allele combinations. Therefore, we focused on generating a mutant population in a pre-green revolution tall cultivar Indian released in the early 1900s.

In wheat, it is relatively easy to identify and recover mutations even in vital genes due to partial compensation by the homoeologs. In hexaploid wheat, about 86% of the genes show significant differences among homoeologs either for the expression level and/or for tissue specificity [15]. These results suggest that most of the hexaploid wheat homoeologs may have diverged to develop subtle differences in their functions thus making it possible to study effect of each copy individually. For some traits such as plant height, peduncle length, tiller number, plant maturity, awns, and leaf spots, a number of mutants showing a range of phenotypes from mild to severe were identified during the present study. These results might be due to complex genetic control of these traits involving multiple genes operating in complex interactions. Part of this phenotypic range is probably due to the involvement of multiple homoeologues corresponding to the underlying genes.

### Succession of mutations over generations

Various concentrations of EMS have been successfully used in wheat to produce mutant populations but an ideal concentration is not known that induces maximum number of mutations per plant while maintaining a higher proportion of fertile plants. Genotypic differences for the response to EMS have also been reported. Furthermore, vendor-to-vendor and batch-to-batch differences in the potency of EMS have also been speculated. After testing a full range of concentrations, 40 mM was identified to be an ideal as it gave the highest proportion of mutant phenotypes. The 50 mM concentration resulted in a higher proportion of aberrant mutants but was not selected as the proportion of fertile mutants was severely reduced than the 40 mM concentration (data not shown).

Different tillers in M_1_ plant are known to be chimeric for mutations and the main tiller is known to have the highest proportion of mutations [16]. Therefore, we planted the M_1_ generation at a very high plant density to ensure only single tiller for each of the M_1_ plants. Redundancy within the mutant population was avoided by taking a single M_2_ seed from the main spikes of each of the M_1_ plants to make the M_2_ generation. In case of heterozygous mutations in a M_2_ seed, only one-fourth of the M_3_ seeds should be homozygous recessive for the mutation(s) thus the probability of losing a mutation in M_3_ generation is small due to a large number of plants sown per mutant line in the field.

A large number of mutations observed during M_2_ generation were not visible at M_3_ generation were thus unstable. Only 5% (24) of the phenotypic and 35% (258) of the height M_2_ mutants were stable at M_3_ generation. Mutants identified in M_3_ generation showed much higher stability during M_4_ generation. About 75% (33) of the phenotypic mutants and 100% (42) of the height mutants were stable during M_4_ generation.

### Enhancement of genetic diversity for wheat breeding

About 95% of the wheat grown in the world contains the same *rht* mutations that are associated with negative impact on seedling emergence and vigor, root biomass, coleoptile, and leaf width [12,11]. Some of these traits are directly linked with plants’ ability to deal with abiotic stresses such as drought and temperature that are expected to become more prominent in the coming years. Therefore, one of our objectives was to identify different types of dwarfing mutations without the ill effects known to be associated with the *rht* mutations. Probably because of our focus, plant height was the most abundant class of mutations accounting for 57% of the total stable mutants identified during this study. In spite of the potential bias for selection, our results suggest that plant height is a complex trait controlled by many different genes. There were significant differences among mutants for the relative reduction in internode length thus it would be possible to select an ideal dwarf plant out of the mutant collection. About 50% of the stable height mutants were very dwarf with plant height ranging from 33 to 59 cm and 21 showed plant height in the range of currently grown wheat varieties thus might have an agronomic value. These mutants are now being evaluated for their GA sensitivity in order to identify height mutants in a pathway other than GA, as GA is known to play an important role in abiotic stress tolerance.

Based on the replication of phenotype and the height of selected mutants in M_4_ generation, we have identified 75 stable M_4_ mutants. These mutants might serve as a useful resource for improving the plant architecture, agronomic performance, and genetic studies. Tiller number controls the number of spikes and is one of the key components in controlling wheat yield. A mutant in rice—*Monoculm* 1 results in single tiller [17]. Its orthologs in Arabidopsis and tomato regulate branching [18,19]. In *Triticum monococcum* subsp. *monococcum*, single tiller mutant named *tiller inhibition* (*tin3*) [20] and *tin* in hexaploid wheat resulted in increased number of kernels per spike [17]. We found a similar uniculm mutant in our mutant population that remained stable over generations. This mutant could serve as a useful resource to understand the underlying mechanism of axillary meristem initiation, bud development, and tiller elongation in wheat.

We have identified a mutant for tiller angle in wheat. The mutant showed hyper-gravitropic response and bends towards soil surface in one direction along with short plant height and the wild type spike length. Similar type of mutants known in other tillering grasses have prostrate growth habit but with tillers spreading in all the directions. Therefore, it is a useful mutant to study the physiological mechanism controlling directional manifestation of the tiller angle. A number of mutants for tiller angle due to the disruption of polar auxin transport and gibberellins biosynthesis have been identified in different plant species including rice, maize, Arabidopsis, and sorghum [21–23]. Additionally, we found a mutant with upright leaves as a potential candidate to modify the plant architecture. Erect leaf architecture reduces shading on the lower leaves and allows better distribution of light to the lower portion of the plant, thus enables a higher plant density [24]. Erect leaf trait has also been shown to be beneficial in maize and rice to increase grain yield [25,26].

Among the identified novel mutants, the physiological mechanisms behind stem bending only in restricted areas such as peduncle and lower internodes are of great importance to study. Stable nature of these mutants over generations eliminates the effect of external signals. Stem bending in plants is known to be regulated through auxin distribution that results in irregular cell division and elongation [27,28]. Therefore, the targeted bending of peduncle and lower internodes in the two newly identified mutants might involve the differential distribution of local auxin hormone. Coleoptile protects the first leaf during emergence until it comes through the soil surface and provides mechanical force for seedling emergence. The gritty coleoptile observed in our population consisting of thick layer of tissue with a white, rough, and sturdy phenotype is an important mutant to study the plant emergence under deep and compact soils.

Additionally, mutants found with phenotypes similar to some of the already identified mutants in other cultivars of wheat may represent an additional set of allelic versions of the corresponding genes. This population can also be a potential resource for physiological screening of diseases as we have phenotypically observed variations for rust resistance in the population (data not shown).

### Detection of mutations through sequencing

In previously reported mutant populations, the frequency of mutations ranged from 1/47 kb to 1/12 kb based on few targeted genes in population size of 1,152 to 2,610 with an EMS concentration of 0.8 to 1% [4,9]. Using Genotyping-by-Sequencing (GBS), EMS induced mutations were identified in a subset of 21 mutants of this population [10]. With 21 mutants and two control Indian plants, 28% of wheat genic sequence was covered by the reads generated by GBS., A total of 14,130 mutational changes [Single Nucleotide Polymorphisms (SNPs) and Insertions/Deletions (INDELs)] identified from 10,374 unigenes corresponding to 72 Mb genic region thus resulted in a frequency of one mutational change per 5 kb of the wheat genome. In addition to the accumulative frequency, a mutation frequency ranging from 1/2.5 kb to 1/6.4 kb was identified from individual mutant plants [10]. Sequence data from this population has been submitted to the National Center for Biotechnology Information (NCBI) Sequence Read Archive (http://www.ncbi.nlm.nih.gov/sra/?term=SRP056743). Wide range of phenotypic mutants and higher number of genomic sequence changes observed in this population establishes it as a valuable resource for forward and reverse genetic studies in wheat.

## Conclusions

The EMS induced mutant population in pre-green revolution cultivar Indian is extensively evaluated and cataloged for different phenotypic traits to increase its utility and is available for the wheat community. Novel mutant phenotypes identified in this population will serve as a useful resource for both genetic and physiological studies in wheat. The mutants identified for desirable agronomic traits can be utilized to transfer these mutations to other cultivars.
